# Correlation between Tooth Position Parameters and Apical Fenestration: A Cone-Beam Computed Tomography Study

**DOI:** 10.3390/mps7010014

**Published:** 2024-02-02

**Authors:** Carlos Henrique Ferrari, Lara Steffany de Carvalho, Caroline Trefiglio Rocha, Amjad Abu Hasna

**Affiliations:** 1 Department of Restorative Dentistry, Endodontics Division, Institute of Science and Technology, São Paulo State University (ICT-UNESP), São José dos Campos 12245-000, SP, Brazilcaroltrefiglio@hotmail.com (C.T.R.); 2 Department of Biosciences and Oral Diagnosis, Institute of Science and Technology, São Paulo State University (ICT-UNESP), São José dos Campos 12245-000, SP, Brazil; lara.s.carvalho@unesp.br; 3 School of Dentistry, Universidad Espíritu Santo, Samborondón 092301, Ecuador

**Keywords:** apical fenestration, cone-beam computed tomography, tooth position

## Abstract

This study aimed to assess the relationship between apical fenestration—a defect in the alveolar bone involving the root apex—and tooth position in all tooth groups, excluding the third molars, utilizing cone-beam computed tomography (CBCT) images. A total of 800 CBCT scans (400 maxillary and 400 mandibular) from patients undergoing various treatments were examined by a single professional (radiologist and endodontist). Statistical analyses, including the chi-square test or Fisher’s exact test, were conducted using R software 2.7.3 (R Foundation, Vienna, Austria). Results indicated a significant association (*p* ≤ 0.05) between apical fenestration and tooth position. In the upper teeth, apical fenestrations were notably present in the mesio-buccal (17.17%) and disto-buccal (11.07%) roots of the first molars. Conversely, apical fenestrations in the lower teeth were relatively less frequent. The study revealed a negative correlation between apical fenestration and mesial inclination, rotation, and extrusion in the upper teeth. However, a positive correlation was observed between apical fenestration and lingual inclination in the upper teeth. In conclusion, this study illuminates the distribution of apical fenestration and its correlation with tooth positions, offering insights into factors influencing this defect in dental anatomy. The findings enhance our understanding of nuanced relationships between tooth position and apical fenestration in the upper and lower dental arches.

## 1. Introduction

Dental fenestration denotes a structural anomaly in the alveolar bone, arising from physiological or procedural factors [1,2,3]. Essentially, it manifests as either a bone window through which a root is exposed or as a deficiency in bone coverage, with only the periosteum and gingival mucosa remaining. In particular cases where the root apex is involved, it is referred to as apical fenestration, signifying the protrusion of the root apex through the external cortical plate [4,5]. This condition, whether caused by natural processes or dental procedures, highlights a disruption in the typical bone structure around teeth. Apical fenestration specifically underscores the involvement of the root apex, emphasizing the significance of these structural irregularities in dental health. Understanding and addressing such manifestations are crucial for comprehensive dental care and treatment planning [6,7]

Apical fenestration may be found in deciduous and permanent teeth [8,9], usually has no symptoms [10], and may be detected during a routine clinical examination [9]. Despite its asymptomatic nature, complications may arise, such as chronic post-operative pain after endodontic procedures, [11] attributed to factors like overfilling [7] or apical extrusion of chemical substances [12]. This underscores the importance of vigilance in dental care, as seemingly benign conditions like apical fenestration can lead to discomfort and complications, necessitating careful management during and after endodontic treatments [7].

Its diagnosis is usually complicated because it may appear radiographically as a radiolucent periapical lesion [13,14], and this is related to the fact that conventional two-dimensional radiography has many limitations [15]. Conversely, cone-beam computed tomography (CBCT) is a three-dimensional scanning method that produces high-resolution images and does not overlap the anatomical structures [16]. This enhances precision in evaluating small alveolar bone defects and their locations. The shift to CBCT signifies a significant improvement in diagnostic capabilities, enabling more accurate assessment of apical fenestration compared to traditional radiographic methods and thereby facilitating better-informed treatment decisions in dental care [17].

Apical fenestration has many predisposing factors, including tooth position (like labial or lingual inclination of the tooth in the alveolar bone), tooth morphology, contour of the root apex, occlusal factors, and others [14,18,19]. The positioning of the tooth plays a significant role, influencing the extent of apical fenestration and diminishing the thickness of the alveolar bone [18,20]. Understanding these predisposing factors is crucial for assessing and managing apical fenestration, as they shed light on the structural considerations that contribute to this dental anomaly.

This study aimed to assess the correlation between the presence of apical fenestration in all tooth groups (excluding the third molars) and tooth position (mesial inclination, lingual inclination, rotation, and extrusion) using CBCT images. The null hypothesis posits a negative correlation between the presence of apical fenestration and tooth position. The investigation focuses on elucidating potential relationships between these factors, providing valuable insights into the anatomical considerations affecting apical fenestration across various tooth groups.

## 2. Materials and Methods

### 2.1. Study Design

This study received approval from the research ethics committee of São Paulo State University (Registration No. 1.079.312) and adhered to the principles outlined in the Helsinki Declaration. All participating patients provided voluntary informed consent, emphasizing ethical considerations and compliance with established research guidelines.

**Figure 1:**
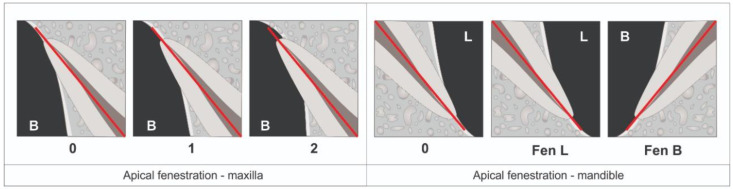
Schematic illustration of the occurrence of apical fenestration. In the maxilla, the score (0) tooth has no apical fenestration; the score (1) tooth has apical fenestration below the buccal cortical plate; and the score (2) tooth has apical fenestration that involves the whole tooth apex (2). In the mandible, the score (0) tooth has no apical fenestration; the score (L) tooth has lingual apical fenestration; and the score (B) tooth has buccal/labial apical fenestration.

The study utilized 800 cone-beam computed tomography (CBCT) scans, evenly distributed between 400 maxillary and 400 mandibular scans, obtained from patients recommended for various dental treatments. Inclusion criteria required participants to be 21 years or older with more than eight teeth in each dental arch. Exclusions included patients with CBCT scans indicating apical resorption, previous root canal treatment, root dilaceration, anomalies, fractures, periapical bone rarefaction, or lacking crowns. Scans with image distortions or technical errors were also excluded. These rigorous criteria ensured a comprehensive and reliable dataset for examining apical exposure beyond the buccal/labial or palatal/lingual cortical plate in the maxillary and mandibular arches, as illustrated in Figure 1.

Images were obtained using a GX CB 500 volumetric CT machine (Gendex/Kavo, Bieberach, Germany) with a 0.20 voxel size, a 14 cm × 8 cm field of view (FOV), 120 kVp, 36.15 mAs, and 12 bits of grayscale depth. Stored in DICOM format, the image data underwent reconstruction using dedicated imaging software (Image Studio 3.4 ^®^ by AnneSolutions, São Paulo, SP, Brazil). This process facilitated the acquisition of standardized sagittal and axial sections at a consistent 1.0 mm interval. Precision was ensured through the involvement of a single professional well versed in radiology and endodontics for all measurements, contributing to the reliability and accuracy of the imaging analysis [14].

### 2.2. Statistical Analysis

Comparisons were conducted in paired samples using the paired *t*-test. Means were compared among three independent groups using ANOVA (analysis of variance). When a considerable deviation from the assumptions for ANOVA application was observed, a non-parametric alternative, the Kruskal–Wallis test, was employed for comparing medians among three independent groups. Subsequently, Dwass–Steel–Critchlow–Fligner tests were used to obtain adjusted *p*-values for multiple comparisons when appropriate.

Quantitative variables with normal and asymmetric distributions were described as mean ± standard deviation or median (interquartile range), respectively. Normality was assessed through visual inspection of histograms and the application of the Shapiro–Wilk normality test. Association tests in contingency tables were conducted using the chi-square test or Fisher’s exact test (and its generalization, the Fisher–Freeman–Halton exact test) when appropriate.

R software 2.7.3 (R Foundation, Vienna, Austria) was utilized for the statistical analysis of the data. All presented *p*-values are two-sided; *p* < 0.05 was considered significant, and 0.05 ≤ *p* ≤ 0.10 was considered marginally significant.

### 2.3. Presence of Apical Fenestration

For both the maxillary and mandibular arches, we focused on sagittal reconstructions that provided a central view of the apices of all teeth. These reconstructions were used to classify the extent of apical exposure beyond the buccal/labial or palatal/lingual cortical plate, as described below (Figure 1):

In the maxilla:

- Score 0 indicates that the tooth exhibits no apical fenestration.
- Score 1 signifies that the tooth displays apical fenestration below the buccal cortical plate.
- Score 2 indicates the presence of apical fenestration that encompasses the entire apex of the tooth.

In the mandible:

- Score 0 denotes the absence of apical fenestration.
- Score L indicates lingual apical fenestration.
- Score B denotes buccal/labial apical fenestration.

### 2.4. Assessment of Tooth Positions

**Figure 2:**
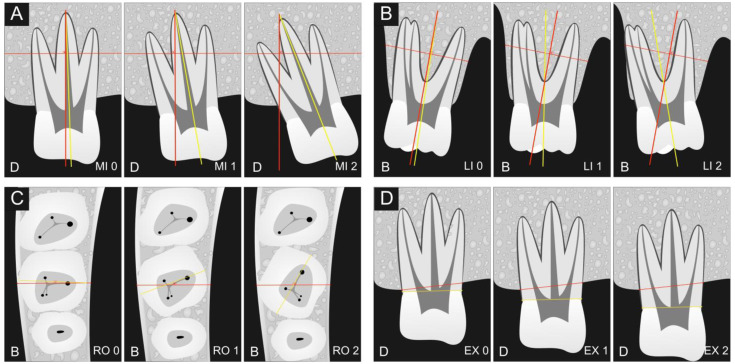
Schematic illustration of the presence of mesial inclination (MI) in subfigure (**A**), lingual inclination (LI) in subfigure (**B**), rotation (RO) in subfigure (**C**), and extrusion (EX) in subfigure (**D**). Legend: the letter D means distal, the letter B means buccal.

Four key parameters were measured to assess the position of all teeth within the dental arch, as outlined below (Figure 2):

(a) Mesial inclination (MI): This parameter was evaluated in the panoramic reconstruction and categorized based on the mesial inclination of the crown as follows:

- MI0 indicates a mesial crown inclination of up to 5 degrees.
- MI1 denotes a mesial crown inclination ranging from 5 to 15 degrees.
- MI2 signifies a mesial crown inclination exceeding 15 degrees.

(b) Lingual inclination (LI): Assessment for lingual inclination was performed in the sagittal reconstruction and classified according to the lingual inclination of the crown:

- LI0 represents a lingual crown inclination of up to 5 degrees.
- LI1 represents a lingual crown inclination ranging from 5 to 15 degrees.
- LI2 represents a lingual crown inclination exceeding 15 degrees.

(c) Rotation (RO): This parameter was evaluated in the axial reconstruction and categorized based on the crown’s rotation concerning a straight line connecting the buccal and lingual midpoints of the cortical plates:

- RO0 indicates a crown rotation of up to 15 degrees.
- RO1 denotes a crown rotation ranging from 15 to 30 degrees.
- RO2 signifies a crown rotation exceeding 30 degrees.

(d) Extrusion (EX): Extrusion measurement was performed in the panoramic reconstruction and classified based on the tooth’s extrusion within the dental arch relative to a straight line connecting the adjacent alveolar crests:

- EX0 signifies no extrusion of the tooth.
- EX1 denotes tooth extrusion of 1–2 mm.
- EX2 indicates tooth extrusion exceeding 2 mm.

Subsequently, all recorded values were tabulated, and statistical analyses were conducted to examine the potential associations between these four tooth position parameters and the presence of apical fenestrations.

## 3. Results

### 3.1. Presence of Apical Fenestration

**Figure 3:**
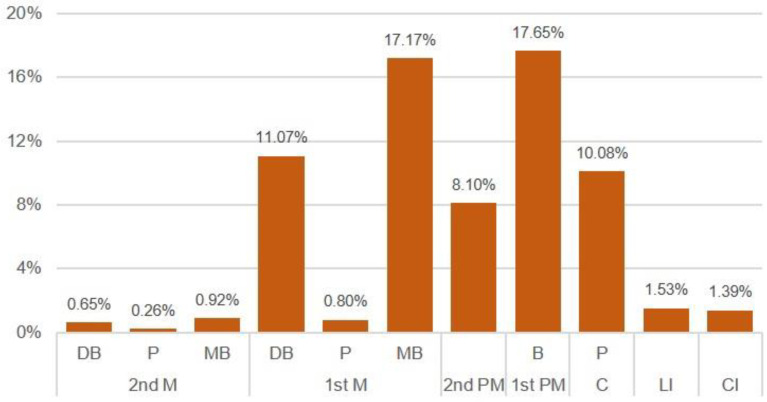
The occurrence of apical fenestration in upper teeth. Legend: M = molar; PM = premolar, C = canine; LI = lateral incisor; and CI = central incisor.

**Figure 4:**
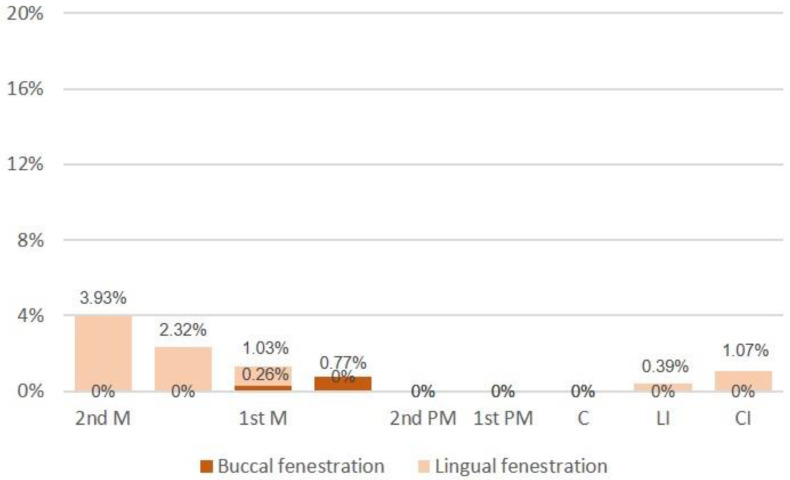
The occurrence of apical fenestration in lower teeth. Legend: M = molar; PM = premolar, C = canine; LI = lateral incisor; and CI = central incisor.

Apical fenestrations were more prevalent in the upper teeth, with notably higher occurrences observed in the mesio-buccal (17.17%) and disto-buccal (11.07%) roots of the first molars. Additionally, apical fenestrations were identified in 17.65% of the upper first premolars, 10.08% of the canines, and 8.10% of the upper second premolars (see Figure 3). In contrast, the presence of apical fenestrations in the lower teeth was comparatively less frequent than in the upper teeth. The highest percentages were noted in the second and first molars, while no apical fenestrations were detected in the lower premolars and canines (refer to Figure 4). These findings delineate distinct patterns in the prevalence of apical fenestrations between the upper and lower teeth, offering valuable insights into dental anatomy and potential clinical considerations.

### 3.2. Assessment of Tooth Positions

Table 1 presents a comprehensive analysis of the relationship between the presence of apical fenestration and various tooth position parameters, including mesial inclination, lingual inclination, rotation, and extrusion. The results indicate a negative correlation in the upper teeth between the presence of apical fenestration and mesial inclination, rotation, and extrusion. Specifically, lower percentages of upper teeth exhibiting these positions were associated with the presence of apical fenestration, reaching statistical significance with scores of MI0, RO0, and EX0 (*p* < 0.0001). Conversely, a positive correlation emerged between the presence of apical fenestration in the upper teeth and lingual inclination. A higher percentage of upper teeth displaying lingual inclination was associated with the presence of apical fenestration, and this correlation achieved statistical significance with a score of LI0 (*p* < 0.0001). These findings offer nuanced insights into the nuanced correlations between apical fenestration and specific tooth positions in the upper dental arch.

In the mandible, no statistically significant differences were observed between the presence of apical fenestration and various tooth position parameters, such as mesial inclination, lingual inclination, rotation, and extrusion (*p* > 0.05) (Table 1). These findings indicate a distinct contrast with the upper dental arch, suggesting that the correlation between apical fenestration and tooth position parameters may vary between the maxillary and mandibular arches. 

## 4. Discussion

This study used CBCT scans because its high quality in the detection and evaluation of small defects of the alveolar bone and their locations is more accurate [15]. Significantly, the investigation unveiled a noteworthy positive association between the occurrence of apical fenestrations and the lingual inclination observed in the upper teeth. This outcome carried substantial implications, as it necessitated the rejection of the null hypothesis, which had initially posited no significant relationship between these variables. The findings thus underscored the importance of CBCT scans in enhancing our understanding of dental conditions and their anatomical underpinnings, particularly in cases where alveolar bone defects and tooth positioning are concerned.

In the broader literature, research from various populations suggests variations in the incidence of apical fenestrations. A classic study [21] that evaluated 2205 teeth in Italian and Austrian males demonstrated that apical fenestrations are more common in the upper teeth than the lower, and that the upper first molars have the highest incidence of apical fenestrations. However, another study found that the incidence of apical fenestrations is more common in the first premolars in cadavers of South African black people [22]. Generally, apical fenestrations are more frequent in the maxilla than in the mandible [23]. The results of the present study agree with the outcomes of all these studies, in which apical fenestrations were more frequent in the first molars and first premolars (Figure 3), in addition to being more frequent in the upper teeth than the lower (Table 1).

In a recent study, it was found that apical fenestrations were more frequent in the buccal surfaces compared to the lingual surfaces in 1189 teeth of an Iranian population [24]. The present research identified a similar pattern, with apical fenestrations being more prevalent in the disto-buccal and mesio-buccal roots of the upper first molars, as well as in the buccal roots of the first premolars. These findings underscore the importance of considering tooth location and root orientation when evaluating the occurrence of apical fenestrations. This insight enhances our understanding of dental anatomy and holds relevance for potential clinical implications in the diagnosis and management of apical fenestrations. 

More recently, in a CBCT study including adolescent and adult patients, it was found in all patients that the maxillary second molar showed buccal inclination, and the mandibular second molar showed lingual inclination [25]. These findings provide a rationale for the occurrence of lingual fenestrations in the lower teeth as identified in the current study. The observed patterns in tooth inclination contribute valuable contextual information, aiding in understanding and explaining the prevalence of specific dental anomalies such as lingual fenestrations in the examined patient population.

The incidence of apical fenestrations is affected by diverse factors, including malocclusion, as a positive correlation was found between apical fenestration and the presence of skeletal Class I, II, and III malocclusions in 123 patients (males and females) in a Turkish population [23], besides other physiological and pathological factors [26]. In the present study, it was found that there was a negative correlation between the presence of apical fenestration and the mesial inclination, rotation, and extrusion of the upper teeth, in which lower percentages of these teeth positions were associated with the presence of apical fenestration in the upper teeth with a statistically significant difference with the scores MI0, RO0, and EX0 (*p* ≤ 0.05). Conversely, there was a positive correlation between the presence of apical fenestration and the lingual inclination in the upper teeth, in which a greater percentage of lingual inclination was associated with the presence of apical fenestration in the upper teeth with a statistically significant difference with the score of LI0 (*p* ≤ 0.05).

Highlighting the significance of apical fenestrations, their association with diverse risk factors is notable, including traumatic injuries, periodontal disease, buccally inclined roots, occlusal trauma, orthodontic interventions, thin alveolar bone overlay, and endodontic pathosis [9,26,27,28]. When apical fenestrations align with endodontic pathology, swift diagnosis and apt management become pivotal for the success of endodontic treatment [26]. Recognizing these risk factors underscores the complexity of factors contributing to fenestration development and emphasizes the importance of tailored intervention strategies. Timely and accurate identification of apical fenestrations, particularly when intertwined with endodontic concerns, facilitates proactive measures, optimizing the overall outcome of endodontic treatments. This comprehensive understanding guides clinicians in addressing the multifaceted nature of apical fenestrations, promoting effective management, and enhancing the success of endodontic interventions.

This study represents a pioneering effort in investigating the relationship between tooth positioning and apical fenestrations, shedding new light on this dental phenomenon. The findings hold valuable implications for clinicians, highlighting the need for heightened awareness regarding the incidence of apical fenestrations in conjunction with the lingual inclination of the upper teeth. Given the potential clinical significance of apical fenestrations, any observed instances should be subject to confirmation through CBCT scans. This preference for CBCT scans is grounded in their well-documented efficacy in diagnosing and executing treatments, as substantiated by previous research [17]. The consequences of misdiagnosis can be severe, leading to repetitive, ineffective treatments and persistent pain, all while jeopardizing the long-term health of the affected tooth [29]. Therefore, this study underscores the importance of precise diagnosis through CBCT scans, offering a more accurate and informed approach to managing cases involving apical fenestrations and their correlation with tooth inclination.

Various injuries, because of chemical, physical, and biological factors included in the root canal treatment [30,31], can impact sensitive anatomical areas [32]. These injuries range in severity from minor issues that are resolved spontaneously to more serious incidents necessitating surgical intervention. Examples include incidents such as sodium hypochlorite overflow, fractures of endodontic instruments, extrusion of debris and obturation cement, and occurrences of labio-mandibular paresthesia [33,34,35,36,37]. Additionally, the concept of apical foramen widening is discussed, allowing for increased chemical and mechanical intrusion in the periapical area during instrumentation. It is crucial to note that while instrumentation is essential, it should not surpass the limits of the apical foramen, and especially in cases of apical fenestrations [26]. 

This study’s clinical applications are impactful. Dentists can enhance diagnostic precision using CBCT scans to assess apical fenestrations, tailoring treatment plans based on the correlation between fenestrations and tooth inclination [38]. The findings inform preventive measures, educational outreach, and patient counseling, fostering a proactive approach to oral health [39]. Regular follow-up with CBCT scans is recommended for monitoring, while this study contributes to refining treatment protocols and inspires further research into the relationship between tooth positioning and apical fenestrations.

In addition, the findings of this study, particularly the positive correlation between apical fenestrations and lingual inclination in the upper teeth, offer valuable insights for orthodontic practitioners. Orthodontists can use this information to anticipate and address potential complications related to apical fenestrations during treatment planning. Understanding the association between tooth inclination and fenestrations allows for more precise orthodontic interventions, potentially minimizing the risk of fenestration development or optimizing management strategies if fenestrations are present. In addition, in the context of endodontic treatment, where apical fenestrations may coincide with pathology, this study emphasizes the need for thorough diagnostic assessments, particularly through CBCT scans. The negative correlation between apical fenestrations and certain tooth positions (mesial inclination, rotation, and extrusion) suggests that specific tooth orientations may be associated with a lower likelihood of fenestration occurrence. Endodontists can use this information to enhance their diagnostic accuracy, tailor treatment plans accordingly, and consider the potential impact of tooth positioning on the success of endodontic interventions. 

## 5. Conclusions

In conclusion, this study establishes a clear and significant positive correlation between the presence of apical fenestrations and lingual inclinations in upper teeth. These findings underscore the importance of considering tooth positioning when assessing the occurrence of apical fenestrations. Clinicians should take this correlation into account in their diagnostic and treatment planning processes. Accurate diagnosis, potentially aided by CBCT scans, is crucial to ensure effective and appropriate treatment, thereby minimizing the risk of repeated interventions and potential complications such as tooth loss. This research contributes valuable insights to our understanding of dental anatomy and offers practical implications for dental practitioners, enhancing their ability to provide optimal care for patients with apical fenestrations associated with lingual inclinations in the upper teeth.

## Figures and Tables

**Table 1:** The correlation between apical fenestrations and the tooth position. Legend: mesial inclination (MI); lingual inclination (LI); rotation (RO); and extrusion (EX).

Maxilla																			
Mesial Inclination					Lingual Inclination					Rotation					Extrusion				
	**0**	**1**	**2**	**Total**		**0**	**1**	**2**	**Total**		**0**	**1**	**2**	**Total**		**0**	**1**	**2**	**Total**
**MI0**	5115	322	64	5501	**LI0**	4587	34	4	4625	**RO0**	5451	340	73	5864	**EX0**	5445	315	69	5829
**MI1**	843	53	30	926	**LI1**	1363	214	45	1622	**RO1**	522	37	24	583	**EX1**	447	61	28	536
**MI2**	52	6	5	63	**LI2**	60	133	50	243	**RO2**	37	4	2	43	**EX2**	118	5	2	125
**Mandible**																			
	**0**	**L**	**B**	**Total**		**0**	**L**	**B**	**Total**		**0**	**L**	**B**	**Total**		**0**	**L**	**B**	**Total**
**DI**	77	0	0	77	**BI**	411	45	2	458										
**MI0**	3813	47	4	3864	**LI0**	3822	5	0	3827	**RO0**	3988	44	2	4034	**EX0**	4442	50	4	4496
**MI1**	466	2	0	468	**LI1**	273	0	2	275	**RO1**	460	6	2	468	**EX1**	65	0	0	65
**MI2**	155	1	0	156	**L12**	5	0	0	5	**RO2**	63	0	0	63	**EX2**	4	0	0	4

## Data Availability

The data used to support the findings of this study are available upon request from the corresponding author.
